# Supplementary material for: Lessons learned from an fMRI-guided rTMS study on performance in a numerical Stroop task
Source: PLoS One. 2024 May 6;19(5):e0302660. doi: 10.1371/journal.pone.0302660 (PMC11073721; doi:10.1371/journal.pone.0302660)
Supplement: S1 Table — Stimulation coordinates are in LPI coordinates system, t-scores at the stimulated target, stimulation intensity expressed as a percentage of the resting motor threshold, Euclidean distance to Sack et al. average target, and scalp-to-cortex distance for each participant. Highlighted in blue are the lowest values and in red the highest values. Participant is italics font was the outlier not included in the analyses. (DOCX) [file pone.0302660.s001.docx]

­­­Lessons learned from an fMRI-guided rTMS study on performance in a numerical Stroop task.

Lysianne Beynel ^1^ ^¶ *^., Hannah Gura^1,2^ ^¶,^ Zeynab Rezaee^1^, Ekaete C. Ekpo^1^, Zhi-De Deng,^1^, Janet, O. Joseph^1,3^, Paul Taylor^4^, Bruce Luber^1^, & Sarah H. Lisanby ^1^.

**S1 Table: Summary of stimulation target location and stimulation intensity for each participant:** Stimulation coordinates are in LPI coordinates system, t-scores at the stimulated target, stimulation intensity expressed as a percentage of the resting motor threshold, Euclidean distance to Sack et al. average target, and scalp-to-cortex distance for each participant. Highlighted in blue are the lowest values and in red the highest values. Participant is italics font was the outlier not included in the analyses.

| Participant | Stimulated target coordinates in MNI space | T score at stimulated target | Intensity (%rMT) | Distance to Sack et al (mm) | Scalp-to-cortex distance (mm) |
| --- | --- | --- | --- | --- | --- |
| Stp_1 | 16.45; -74.79; 61.52 | 1.46 | 118 | 19 | 25.3 |
| Stp_3 | 32.74; -46.50; 73.39 | 2.35 | 115 | 41 | 26.4 |
| Stp_5 | 23.71; -75.19; 58.54 | 1.86 | 154 | 16 | 21.5 |
| Stp_6 | 22.57; -63.64; 69.11 | 2.03 | 125 | 26 | 26.7 |
| Stp_7 | 16.09; -69.80; 65.99 | 2.50 | 107 | 24 | 29.2 |
| Stp_9 | 34.73; -66.64; 54.89 | 1.81 | 136 | 19 | 20.5 |
| *Stp_10* | *36.70; -83.90; 35.50* | *1.49* | *94* | *21* | *30.4* |
| Stp_12 | 35.82; -40.59; 71.61 | 2.02 | 130 | 45 | 22.4 |
| Stp_13 | 30.59; -86. 99; 37.61 | 2.06 | 188 | 19 | 20.4 |
| Stp_14 | 29.31; -67.84; 62.71 | 1.10 | 122 | 22 | 21.0 |
| Stp_15 | 30.39; -71.55; 61.40 | 2.03 | 136 | 21 | 15.8 |
| Stp_16 | 39.49; -63.31; 60.21 | 2.14 | 111 | 27 | 22.5 |
| Stp_17 | 36.00; -86.22; 33.76 | 1.91 | 140 | 23 | 19.0 |
| Stp_18 | 47.77; -60.81; 54.95 | 2.33 | 122 | 31 | 27.0 |
| Stp_19 | 19.88; -63.94; 69.22 | 1.96 | 136 | 37 | 18.6 |
|  | Mean ± SE: | 1.94 | 128.93 | 26.06 | 23.11 |
