# Supplementary material for: Lessons learned from an fMRI-guided rTMS study on performance in a numerical Stroop task
Source: PLoS One. 2024 May 6;19(5):e0302660. doi: 10.1371/journal.pone.0302660 (PMC11073721; doi:10.1371/journal.pone.0302660)
Supplement: S2 Table — (DOCX) [file pone.0302660.s002.docx]

­­­Lessons learned from an fMRI-guided rTMS study on performance in a numerical Stroop task.

Lysianne Beynel ^1^ ^¶ *^., Hannah Gura^1,2^ ^¶,^ Zeynab Rezaee^1^, Ekaete C. Ekpo^1^, Zhi-De Deng,^1^, Janet, O. Joseph^1,3^, Paul Taylor^4^, Bruce Luber^1^, & Sarah H. Lisanby ^1^.

**S2 Table**: **Overlap between activated clusters in the Incongruent > Congruent contrast and their overlap with the Glasser atlas**.

| Clust Nvox Overlap ROI location |
| --- |
| 1 160 32.5% L_Area_PFm_Complex |
| 22.6% L_Area_PGi |
| 11.8% L_Area_PGs |
| 2 157 9.7% L_Anterior_24_prime |
| 3 87 6.8% R_Primary_Visual_Cortex |
| 4 83 20.5% R_Parieto-Occipital_Sulcus_Area_2 |
| 14.1% L_Medial_Area_7P |
| 13.6% R_Area_7m |
| 5 68 33.2% L_Area_anterior_47r |
| 17.6% L_Area_anterior_9-46v |
| 6 56 2.8% L_RetroSplenial_Complex |
| 7 56 47.3% R_Area_PGs |
| 30.0% R_Area_PFm_Complex |
| 8 55 42.9% L_Area_a24 |
| 22.0% L_Area_p32 |
| 11.5% L_Area_10r |
| 9 48 32.7% R_Area_9_Posterior |
| 16.6% R_Area_9_anterior |
| 16.2% R_Area_9_Middle |
| 10 41 22.6% L_Hippocampus |
| 12 38 32.0% R_Area_IFSp |
| 17.5% R_Area_45 |
| 13.3% R_Area_44 |
| 14 32 27.8% R_Area_PGi |
| 16 29 31.4% R_Area_10d |
| 17 26 44.0% L_Area_31p_ventral |
| 16.6% L_Area_31a |
| 18 25 1.7% L_Area_Posterior_Insular_1 |
| 19 25 70.8% L_Area_8C |
| 17.6% L_Area_46 |
| 20 24 20.6% R_posterior_OFC_Complex |
| 21 23 27.7% L_Area_Lateral_IntraParietal_ventral |
| 26 21 5.5% R_Fourth_Visual_Area |
| 27 21 37.5% R_Area_posterior_47r |
| 19.9% R_Area_anterior_9-46v |
| 14.0% R_Area_anterior_47r |
| 28 21 81.6% L_Area_8Ad |
| 29 21 43.9% R_Area_8B_Lateral |
| 33 20 36.4% L_Area_11l |
| 34 20 29.5% L_Area_8Av |
| 23.5% L_Inferior_6-8_Transitional_Area |
| 13.7% L_Area_6m_anterior |
