# Supplementary material for: Lessons learned from an fMRI-guided rTMS study on performance in a numerical Stroop task
Source: PLoS One. 2024 May 6;19(5):e0302660. doi: 10.1371/journal.pone.0302660 (PMC11073721; doi:10.1371/journal.pone.0302660)
Supplement: Attachment — Submitted filename: PLOS_One_Review.docx [file pone.0302660.s003.docx]

The present study attempted to replicate prior findings showing that short bursts of high frequency TMS delivered to the right intraparietal sulcus disrupt the Stroop effect (i.e., Cohen Kadosh et al., 2007; Sack et al., 2009). Well conducted replication efforts like this one are rare, at least as can be determined by the published literature. However, I have several questions, comments, and suggested revisions that I think could strengthen the paper.

(1) Was a power analysis performed? If not, the authors should justify their sample size and acknowledge the lack of a power analysis as a limitation. I am skeptical of the authors’ claim that “rTMS effects on task performance are typically seen using sample sizes of thirteen and up”. This claim was made on the basis of their review of a heterogeneous behavioral TMS literature using a variety of TMS protocols and cognitive tasks. Surely, protocol and task differences impact power considerations.
(2) Was the decision to perform drift diffusion models made post-hoc? If so, consistent with the spirit of reproducibility that motivated the present study, it should probably be described that way. I was able to find general study details on clinical trials.gov but did not see a description of planned analyses.
(3) The Discussion includes material that should probably be outlined in the Introduction and better detailed in Methods. For example, drift diffusion models are mentioned only briefly in the Introduction. It would be useful to briefly outline why this approach was used versus others. More importantly, as described in detail in the Discussion section, there are several ways in which the present study differs from the studies that it sought to replicate. It would be useful to preview what motivated these differences in the Introduction and cover them in more detail in Methods.
(4) I think it would be worth testing to see if the ANOVA homoscedasticity assumption is violated, as the congruent/incongruent Stroop conditions may have different variances. If this assumption is not met, mixed models would be a more appropriate way to analyze these data.
(5) It may be worth mentioning (although I think this should be up to the authors) that causally derived targets may be better candidates than fMRI correlates, as these correlates may be spurious or reflect compensatory processes. TMS accessible cortical areas that are strongly connected to TMS/DBS sites or lesions that alter Stroop performance or Stroop relevant processes may be particularly promising.
(6) Could the authors please include a Methods figure detailing study procedures by visit?
(7) I am curious about the authors’ speculations on their drift rate finding that TMS affected congruent versus incongruent trials. This seems worth briefly commenting on in the Discussion section. However, I leave this up to the authors if they feel the speculations are too speculative.
