# Supplementary material for: Lessons learned from an fMRI-guided rTMS study on performance in a numerical Stroop task
Source: PLoS One. 2024 May 6;19(5):e0302660. doi: 10.1371/journal.pone.0302660 (PMC11073721; doi:10.1371/journal.pone.0302660)
Supplement: Attachment — Submitted filename: Rebuttal_Letter.docx [file pone.0302660.s004.docx]

Table of Contents

[Journal requirements:	2](#_Toc159850064)

[Reviewer #1:	5](#_Toc159850065)

[Reviewer #2:	10](#_Toc159850066)

[Reviewer #3:	15](#_Toc159850067)

[Reviewer #4:	21](#_Toc159850068)

[Reviewer #5:	26](#_Toc159850069)

[References:	32](#_Toc159850070)

# ­­Journal requirements:

1. When submitting your revision, we need you to address these additional requirements.

Please ensure that your manuscript meets PLOS ONE's style requirements, including those for file naming. The PLOS ONE style templates can be found at 
https://journals.plos.org/plosone/s/file?id=wjVg/PLOSOne_formatting_sample_main_body.pdf and 
<https://journals.plos.org/plosone/s/file?id=ba62/PLOSOne_formatting_sample_title_authors_affiliations.pdf>.

**Response: The manuscript has been edited to follow Plos one style templates.**

2. Thank you for stating the following financial disclosure: 
 [Beynel L., Gura, H., Rezaee, Z., Ekpo, E., Joseph, J., Deng, Z-D., Luber, B., and Lisanby, S.H. are supported by the NIMH Intramural Research Program (ZIAMH002955).
Taylor, P. was supported by the NIMH Intramural Research Program (ZICMH002888) of the NIH/HHS, USA.].  
Please state what role the funders took in the study.  If the funders had no role, please state: ""The funders had no role in study design, data collection and analysis, decision to publish, or preparation of the manuscript."" 
If this statement is not correct you must amend it as needed. 
Please include this amended Role of Funder statement in your cover letter; we will change the online submission form on your behalf.

**Response: This information has been updated in our cover letter: “The funders had no role in study design, data collection and analysis, decision to publish, or preparation of the manuscript**

3. PLOS requires an ORCID iD for the corresponding author in Editorial Manager on papers submitted after December 6th, 2016. Please ensure that you have an ORCID iD and that it is validated in Editorial Manager. To do this, go to ‘Update my Information’ (in the upper left-hand corner of the main menu), and click on the Fetch/Validate link next to the ORCID field. This will take you to the ORCID site and allow you to create a new iD or authenticate a pre-existing iD in Editorial Manager. Please see the following video for instructions on linking an ORCID iD to your Editorial Manager account: <https://www.youtube.com/watch?v=_xcclfuvtxQ>".

**Response: The corresponding author ORCID ID has been added to the Editorial Manager**

4. Thank you for stating the following in the Competing Interests section: 
[The opinions expressed in this article are the authors' and do not reflect the views of the National Institutes of Health, the Department of Health and Human Services, or the United States government.  Dr. Deng is inventor on patents and patent applications on electrical and magnetic brain stimulation therapy systems held by the National Institutes of Health (NIH), Columbia University, and University of New Mexico. Dr. Lisanby is inventor on patents and patent applications on electrical and magnetic brain stimulation therapy systems held by the NIH and Columbia University, with no remuneration.].  Please confirm that this does not alter your adherence to all PLOS ONE policies on sharing data and materials, by including the following statement: ""This does not alter our adherence to PLOS ONE policies on sharing data and materials.” (as detailed online in our guide for authors http://journals.plos.org/plosone/s/competing-interests).  If there are restrictions on sharing of data and/or materials, please state these. Please note that we cannot proceed with consideration of your article until this information has been declared. Please include your updated Competing Interests statement in your cover letter; we will change the online submission form on your behalf.

**Response: We have updated the competing interest’s statement in our cover letter.**

5. Thank you for uploading your study's underlying data set. Unfortunately, the repository you have noted in your Data Availability statement does not qualify as an acceptable data repository according to PLOS's standards. At this time, please upload the minimal data set necessary to replicate your study's findings to a stable, public repository (such as figshare or Dryad) and provide us with the relevant URLs, DOIs, or accession numbers that may be used to access these data. For a list of recommended repositories and additional information on PLOS standards for data deposition, please see <https://journals.plos.org/plosone/s/recommended-repositories>.

**Response: Data have been added to “figshare” repository with this URL: https://figshare.com/account/home#/projects/194468**

6. Please include the reference section of your manuscript.

**Response: References were at the end of the manuscript. We will add it separately in the document upload.**

7. We note that Figure(s) 4A and 4B in your submission contain copyrighted images. All PLOS content is published under the Creative Commons Attribution License (CC BY 4.0), which means that the manuscript, images, and Supporting Information files will be freely available online, and any third party is permitted to access, download, copy, distribute, and use these materials in any way, even commercially, with proper attribution. For more information, see our copyright guidelines: <http://journals.plos.org/plosone/s/licenses-and-copyright>. We require you to either (1) present written permission from the copyright holder to publish these figures specifically under the CC BY 4.0 license, or (2) remove the figures from your submission:

 
a. You may seek permission from the original copyright holder of Figure(s) 4A and 4B to publish the content specifically under the CC BY 4.0 license. We recommend that you contact the original copyright holder with the Content Permission Form (http://journals.plos.org/plosone/s/file?id=7c09/content-permission-form.pdf) and the following text:“I request permission for the open-access journal PLOS ONE to publish XXX under the Creative Commons Attribution License (CCAL) CC BY 4.0 (http://creativecommons.org/licenses/by/4.0/). Please be aware that this license allows unrestricted use and distribution, even commercially, by third parties. Please reply and provide explicit written permission to publish XXX under a CC BY license and complete the attached form.” Please upload the completed Content Permission Form or other proof of granted permissions as an ""Other"" file with your submission.  In the figure caption of the copyrighted figure, please include the following text: “Reprinted from [ref] under a CC BY license, with permission from [name of publisher], original copyright [original copyright year].”

b. If you are unable to obtain permission from the original copyright holder to publish these figures under the CC BY 4.0 license or if the copyright holder’s requirements are incompatible with the CC BY 4.0 license, please either i) remove the figure or ii) supply a replacement figure that complies with the CC BY 4.0 license. Please check copyright information on all replacement figures and update the figure caption with source information. If applicable, please specify in the figure caption text when a figure is similar but not identical to the original image and is therefore for illustrative purposes only.

[Note: HTML markup is below. Please do not edit.]

**Response: Figure 4 is our own figure, no copyright needed.**

# Reviewer #1:

I really enjoyed reading this paper. The authors did a fantastic and rally went the extra mile in terms of methodology and data analyses. Chapeau! I also love that they were inspired by our original article from 2009 and aimed at replicating our comparison of different TMS coil positioning approaches. The discussion is comprehensive and addresses many factors that may have contributed to the result(s) and may explain differences between their and our own findings from 2009. There is so much food for thought in there that I absolutely applaud such as the question of replicability and robustness of TMS-induced behavioral performance effects and their dependence on, e.g., degree of practice on the task at hand, or experimental design choices such as between versus within-subject comparisons etc. This is all wonderful.

**Response: Thank you very much (Merci!) Dr. Sack for those good words on our manuscript, we are glad that you found it interesting and, in the section below we provide a point-by-point response to your concerns.**

1. My only and main concern here is that the title, abstract and introduction, in fact the overall framing of this paper, is misleading and unjustified. The title literally promises to address the question “What is the best way to target rTMS?” And then the abstract and introduction frames this as a replication of our comparison of different targeting approaches from 2009. But, in sharp contrast to this framing, in the current study no comparison of targeting approaches is presented. In fact, only fMRI-guided TMS neuronavigation is used to test for effects on performances in the numerical stroop task. The current study only compares active and sham stimulation and fails to replicate the behavioral RT effects reported in earlier TMS studies using the same task. There is no comparison of TMS coil positioning approaches and the question in the title “What is the best way to target rTMS?” is not addressed by the study. I understand that replicating the behavioral effect is a step towards a replication study of the localization differences reported by us, but now readers will get the impression that they already did that and failed.

**Response: Thank you for raising this important concern. We acknowledge that our initial framing may have created a misleading impression regarding the scope of our study. While our ultimate goal with this research is to compare the efficacy of new targeting approaches, as you say we first wanted to ensure we could produce a reliable behavioral effect on the numerical Stroop task using TMS. Consequently, we decided to rewrite our manuscript so that the framing reflects the scope and methodology of our research.**

**The new title is: “Lessons learned from an fMRI-guided rTMS study on performance in a numerical Stroop task.”**

**The abstract now reads:** “**The Stroop task is a well-established tool to investigate the influence of competing visual categories on decision making. Neuroimaging as well as rTMS studies have demonstrated the involvement of parietal structures, particularly the intraparietal sulcus (IPS), in this task. Given its reliability, the numerical Stroop task was used to compare the effects of different TMS targeting approaches by Sack and colleagues, who elegantly demonstrated the superiority of individualized fMRI targeting. We performed the present study to test whether fMRI-guided rTMS effects on numerical Stroop task performance could still be observed while using more advanced techniques that have emerged in the last decade (e.g., electrical sham, robotic coil holder system, etc). To do so we used a traditional reaction time analysis and we performed, post-hoc, a more advanced comprehensive drift diffusion modeling approach. Fifteen participants performed the numerical Stroop task while active or sham 10 Hz rTMS was applied over the region of the right intraparietal sulcus (IPS) showing the strongest functional activation in the Incongruent > Congruent contrast. This target was determined based on individualized fMRI data collected during a separate session. Contrary to our assumption, the classical reaction time analysis did not show any superiority of active rTMS over sham, probably due to confounds such as potential cumulative rTMS effects, and the effect of practice. However, the modeling approach revealed a robust effect of rTMS on the drift rate variable, suggesting differential processing of congruent and incongruent properties in perceptual decision-making, and, more generally, illustrating that more advanced computational analysis of performance data can elucidate the effects of rTMS on the brain where simpler methods may not.” (PAGE 2)**

**We modified the introduction as follows: “Sack et al. (2009) concluded that the most effective approach was individualized targeting based on the task-related fMRI. This was an important result for the brain stimulation field in general, as one of the main moderators of rTMS efficacy is the targeting approach (Beynel et al., 2019). More than a decade after this elegant demonstration of the importance of individualized fMRI targeting, experimental TMS methodologies have advanced, with for example the development of robotic coil holders that allow for more precise and stable positioning of the coil relative to the stimulation target, and an electrical sham technique that mimics the TMS-induced sensations, and thus potentially better blinds subjects to experimental conditions. In the present study, we tested whether the addition of these techniques combined with an individualized fMRI-based targeting approach endorsed by Sack and colleagues could improve the rTMS effect on numerical Stroop performance, with the expectation based on Cohen Kadosh et al. (2007) and Sack et al. (2009) that rTMS applied over the IPS would decrease the interference of incongruent stimuli and thus lessen of the reaction time slowing in that condition. Additionally, contrary to the prior study from Sack et al., participants completed the task multiple times preceding rTMS application (one block of trials before the MRI session, 4 blocks during MRI, and one block in the TMS session prior to TMS), to stabilize performance and minimize variability due to practice effects. This approach enabled us to probe the influence of task practice on behavioral performance and fMRI activations. (PAGE: 5-6)**

2. The authors seem to suggest that the failed replication of the behavioral effect using fMRI-guided TMS casts doubt on the localization / TMS targeting effect. I think this conclusion is very questionable. The main gist and focus of our study in 2009 were to compare the relative differences between different TMS targeting approaches. It was not meant to claim that you need only 5 subjects or 15 subjects or whatever number of subjects to find a significant behavioral finding in the numerical stroop test when using this or that targeting approach. The design and overall extremely low sample size was completely unsuited for that (5 subjects, between subject design). We were also not interested in the numerical stroop task per se. This task just served as a tool, a means to compare the performance differences induced by the various TMS targeting approaches we compared with each other, showing relative differences. The numerical stroop task we used was brought to us by our visiting researcher, Dr. Roi Cohen Kadosh, who was PI of a research line using this task in the fMRI. In related publications, e.g. Cohen Kadosh 2007, this team reported right parietal activity changed during the execution of this numerical stroop task and behavioral effects when stimulating those individual right parietal activity clusters using TMS. I understand that the authors originally wanted to replicate our targeting approach study but got stopped at step 1, when hoping to first find the expected behavioral changes when using the supposedly best targeting approach: individual fMRI-guided TMS. Since they could not find any behavioral effects here, they now publish this still in the framing of an intended and failed replication of our TMS targeting study. But in fact, they failed to find the behavioral effects using one targeting approach. This is something completely different. In fact, when looking at their results in more detail, the issue of replicating previous work already starts with the fMRI data itself. Unlike previous work by Cohen Kadosh and others, the fMRI data of the current does not demonstrate clear individual right parietal activity clusters to underlie the execution of this task. In fact, in many cases, their fMRI data, if really used to localize the individual hotspot of this task for TMS targeting, would have suggested areas in the left parietal cortex. So, ironically, because they aimed to replicate our study of 2009, they wanted to stick to the right IPS (because this is where we fund our fMRI hotspot in each participant for this task), but by doing so, they actually violated the entire principle of individual fMRI-guided TMS targeting which bases the TMS positioning on the individual fMRI hotspot, data-driven, not hypothesis- or replication-driven. They should have listed the individual fMRI findings and stimulate at the fMRI- based hotspot, and maybe, if they then would have found a significant behavioral effect, they could have compared this to other targeting approaches. This would have been closer to replicating our study than sticking to a right IPS cluster because of an a priori principle. Redo the analyses, without masks or restrictions and do the TMS based on the individual hotspot, no matter whether right or left IPS. Otherwise, you are not capitalizing on the power of individual fMRI guided TMS.

**Response: We appreciate the insightful feedback. While we agree that the principle of individual fMRI-guided TMS targeting is data-driven (and specifically fMRI hotspot-driven), it needs be constrained to a certain extent. With most behavioral tasks, quite a few hotspots across the brain are usually found, and these may be due to various perceptual, motor, and executive processes more (and less) functionally related to carrying out the task. This situation creates a trade-off between freely allowing fMRI hotspots to guide target choice (which, when looking over a large enough range, might result in the choice of a target in one individual that has to do with one process involved in the task and another target in another individual that has to do with a different task-related process) and narrowing in on the same process across individuals. Our solution (used by others as well): If there is prior evidence of a brain region that is related to some sub-process of a task that is the particular interest of the study, that region is probably the best place to focus on initially. Some estimate of the individual variability in the "location" of the functional activity in that region should be used to create a mask within which to locate a hotspot for each individual. In this study, we decided to use the region defined by two papers from your group: Cohen-Kadosh et al. and Sack et al. as an initial mask. In Cohen-Kadosh, while bilateral parietal fMRI activation was found, the TMS effect was observed only when the right hemisphere was stimulated. In Sack et al. only the right hemisphere was stimulated probably based on the finding of Cohen-Kadosh. We therefore followed this line of findings- while we expected bilateral parietal fMRI activation, we targeted the right side. Therefore, we did not violate the entire principle of individualized fMRI targeting, but in fact followed it, based on the previous research (which twice found a right sided TMS effect, but did not find one on the left).**

**However, the lack of a group activation in the right IPS does not necessary imply that it was not a good target. Indeed, it has been demonstrated that fMRI group analysis might not always detect the brain regions that are necessary at the individual level, and instead consider the individual topographic variability as noise; and that applying rTMS on this subject-specific regions, even though variable, can lead to a significant rTMS effect while stimulating the group fMRI activation site does not (Feredoes et al., 2007). This has been added into the discussion section of our manuscript (PAGE 31)**

**Further, we performed an additional analysis comparing activation in the first two fMRI blocks recorded during the task with the second two blocks, and demonstrated there was a dramatic drop in functional activation from an initially significant right IPS activation. This decrease could have resulted from practice, learning, and/or fatigue, and led to a non-significant activation when combining all 4 blocks. As this provides some insight into targeting issues that may have hindered us from finding an rTMS effect, we have included this additional analysis in the manuscript:**

**“To evaluate whether the lack of an rTMS effect may have been due to targeting issues resulting from a change in fMRI activation across blocks, which may have resulted from practice, learning, and/or fatigue, we investigated how activations differed between the first and last pairs of blocks. Our results showed that while activation in the bilateral IPS was strong during the first two blocks, IPS activity vanished during the last two blocks (Figure 6). Given that these changes were observed while accuracy remained quite high and constant across all blocks, and while reaction time continued to improve (Figure 2A), we suspect this change is due to a learning effect and hypothesize two potential mechanisms: 1) the IPS was highly involved in the earlier learning stages of the task and became less necessary with practice, with possible activity shifts to other regions; 2) the number of IPS neurons necessary for task performance decreased with the optimization caused by practice, with subsequent diminishment of signal. If correct, these hypotheses indicate the need for a better understanding of the local and network changes associated with task practice and learning effects to estimate the best targeting parameters. In the present case we potentially would have seen stronger rTMS effects if we only acquired two blocks of fMRI, and this may have served as a better target for rTMS if the latter of the two reasons stated above for the activation decrease was true.” (PAGE 19)**

3. In sum, great study, many lessons learned, great discussion, but title, and framing as a replication of our study from 2009 is misleading, and the study is really not a replication of our work as it differs in too many methodological aspects and doesn’t even include any comparison of different TMS targeting approaches. This can easily be fixed by changing title and reframing abstract and introduction. But I want to again congratulate the authors for conducting this study, I want to encourage them to continue on this path and express my admiration for their high scientific standards and methodological rigor.

**Response: Thank you again for the suggestions, we hope that the revised version of the manuscript will address the concerns raised and improve overall clarity and accuracy.**

Best wishes,
Alexander Sack

# Reviewer #2:

The present study attempted to replicate prior findings showing that short bursts of high frequency TMS delivered to the right intraparietal sulcus disrupt the Stroop effect (i.e., Cohen Kadosh et al., 2007; Sack et al., 2009). Well conducted replication efforts like this one are rare, at least as can be determined by the published literature. However, I have several questions, comments, and suggested revisions that I think could strengthen the paper.

**Response: We would like to thank Reviewer 2 for their useful comments on our manuscript, and we believe following them has improved its quality. We provide below a point-by-point response to each of them.**

(1) Was a power analysis performed? If not, the authors should justify their sample size and acknowledge the lack of a power analysis as a limitation. I am skeptical of the authors’ claim that “rTMS effects on task performance are typically seen using sample sizes of thirteen and up”. This claim was made on the basis of their review of a heterogeneous behavioral TMS literature using a variety of TMS protocols and cognitive tasks. Surely, protocol and task differences impact power considerations.

**Response: Thank you for raising that concern. The reviewer is correct that no power analysis was performed for this study. We based our sample size selection on TMS effects found in previous research using the same task and brain target. Since both Cohen-Kadosh et al. (2007) and Sack et al. (2009) each found significant rTMS effects on numerical Stroop task performance with five subjects, and in fact the results of Sack et al. were presented as a power analysis suggesting five subjects was sufficient to produce a significant (p<.05) effect when using individualized fMRI-based targeting, we expected our larger sample size would be sufficient as well. However, given the changes we made to the design, this clearly was not enough. We added this in the limitation section of our revised manuscript:**

**“In the present study, we tried to adhere closely to the procedures Sack et al. (2009) and Cohen Kadosh et al. (2007) used but were not able to replicate their findings despite a larger sample size. In designing our experiment, we did not initially perform a power analysis on the optimal number of participants needed to observe significant difference between active and sham rTMS, relying on the findings of Cohen-Kadosh et al. and Sack et al., who each found a significant TMS effect with 5 participants. While we tried to emulate their methods, the changes we did make in the experimental design may have potentially lowered the effect size, and we may have needed to employ a larger sample.” (PAGE 34).**

(2) Was the decision to perform drift diffusion models made post-hoc? If so, consistent with the spirit of reproducibility that motivated the present study, it should probably be described that way. I was able to find general study details on clinical trials.gov but did not see a description of planned analyses.

**Response: Yes,­ the analysis of the drift diffusion model was made post-hoc. We edited that in the new version of our manuscript, in the abstract: “To do so we used a traditional reaction time analysis and we performed, post-hoc, a more advanced comprehensive drift diffusion modeling approach. (PAGE 2)**

**and in the introduction: “Finally, we decided post-hoc to perform, a behavioral modeling approach using a drift diffusion model to assess rTMS effects within a decision-making context.” (PAGE 6)**

(3) The Discussion includes material that should probably be outlined in the Introduction and better detailed in Methods. For example, drift diffusion models are mentioned only briefly in the Introduction. It would be useful to briefly outline why this approach was used versus others. More importantly, as described in detail in the Discussion section, there are several ways in which the present study differs from the studies that it sought to replicate. It would be useful to preview what motivated these differences in the Introduction and cover them in more detail in Methods.

**Response: Thank you for raising this concern, we have edited and reframed our introduction section by highlighting that our study differs from the original one and made it the main point. The Introduction now reads as:**

**“Sack et al. (2009) concluded that the most effective approach was individualized targeting based on the task-related fMRI. This was an important result for the brain stimulation field in general, as one of the main moderators of rTMS efficacy is the targeting approach (Beynel et al., 2019). More than a decade after this elegant demonstration of the importance of individualized fMRI targeting, experimental TMS methodologies have advanced, with for example the development of robotic coil holders that allows for more precise and stable positioning of the coil relative to the stimulation target, and an electric sham technique that mimics TMS-induced sensations, and thus potentially better blinds subject to experimental conditions. In the present study, we tested whether the addition of these techniques combined with the individual fMRI-based targeting approach endorsed by Sack and colleagues could improve the rTMS effect on numerical Stroop performance, with the expectation based on Cohen Kadosh et al. (2007) and Sack et al. (2009) that rTMS applied over the IPS would decrease the interference of incongruent stimuli and thus lessen of the reaction time slowing in that condition. Additionally, contrary to the prior study from Sack et al., participants completed the task multiple times preceding rTMS application (one block of trials before the MRI session, 4 blocks during MRI, and one block in the TMS session prior to TMS), to stabilize performance and minimize variability due to practice effects. This approach enabled us to probe the influence of task practice on behavioral performance and fMRI activations. Finally, we decided post-hoc to perform a behavioral modeling approach using a drift diffusion model to assess rTMS effects within a decision-making context. This model, by considering the distribution of reaction times rather than just central tendencies, offers more insight in decision making processes. By elucidating the dynamics of evidence accumulation over time, and by distinguishing between decision and non-decision processes, the model provides a more nuanced understanding of ongoing cognitive mechanisms. Drift diffusion models have demonstrated effectiveness in modeling performance data derived from simple tasks with short reaction times (< 1 s), as in the present case. In addition, they have been linked to neural behavior on a number of scales, from single cells to large populations (e.g., using EEG and fMRI), also highly relevant to the present study (Ratcliff, Smith, Brown, and McKoon, 2016). The drift diffusion models have also been successfully used to investigate the neuronal substrates for numerical representation using TMS (Cohen-Kadosh et al., 2010). Therefore, it was expected that this modeling approach would reveal more subtle rTMS effects, as recommended by Hartwigsen et al. (2015). (PAGE 5-6)**

(4) I think it would be worth testing to see if the ANOVA homoscedasticity assumption is violated, as the congruent/incongruent Stroop conditions may have different variances. If this assumption is not met, mixed models would be a more appropriate way to analyze these data.

**Response: The assumptions of normality, sphericity and homoscedasticity were tested before performing the ANOVA and all were respected (ps > 0.05). However, we decided to replace our ANOVA by a linear mixed model, to provide more appropriate analyses and mitigate potential issues arising from non-independent observations, as highlighted by Reviewer 2.**

**The results section now reads as follows: “A linear mixed model was performed with participants as a random effect, and Stimulation, Congruency and Order as fixed effects. The model revealed a main effect of Congruency (F(2, 25.30) = 47.56, p < 0.001), with incongruent trials slowing down participants (492 ± 15 ms) compared to congruent (439 ± 12 ms) and neutral trials (457 ms ± 12 ms) (p < 0.01 for each pair-wise comparison). The model also revealed a significant interaction with Stimulation, Congruency and Order (F(2, 44.43) = 3.68; p = 0.03). However, decomposition of this interaction with post-hoc Bonferroni- corrected t-test did not reveal any significant difference between active and sham rTMS. (Page 15)**

**For the same reason, we decided to use a linear model to investigate the effect of practice on reaction time: “We assessed the effects of Congruency and Timepoint on correct reaction times during practice using a linear mixed model with Congruent and Timepoint as fixed effect variables, and Participant as random effect. The linear mixed model did not show any interaction between Congruency and Timepoint, however we found the expected main effect of Congruency (F(1,12.58) = 94.698, p < 0.001) with congruent trials yielding faster reaction times (533 ± 16 ms) than incongruent trials (607 ms ± 14 ms). A main effect of Timepoint (F(5, 14.68) = 23.830, p < 0.001) was also observed; and post-hoc contrasts (Bonferroni corrected) revealed that while performance in the pre-MRI practice block was not significantly different from the first MRI block (p > 0.05); reaction times at each of the timepoints thereafter were faster than the initial reaction time collected before the MRI (p < 0.05 for all pair-wise comparisons). Reaction times at all timepoints after MRI block 1 were also significantly faster than for MRI block 1 (p < 0.05 for all pair-wise comparisons). No significant pairwise differences in RT between other blocks were observed (Figure 2A). (PAGE 14)**

(5) It may be worth mentioning (although I think this should be up to the authors) that causally derived targets may be better candidates than fMRI correlates, as these correlates may be spurious or reflect compensatory processes. TMS accessible cortical areas that are strongly connected to TMS/DBS sites or lesions that alter Stroop performance or Stroop relevant processes may be particularly promising.

**Response: We agree with the reviewer that causally derived targets such as DBS sites or brain lesions could be another alternative to the fMRI targeting approach, although it should be remarked that we did employ a causally-based approach to constrain our fMRI targeting, namely, two previous TMS studies that had provided causal evidence that right parietal cortex was involved with numerical Stroop task processing. We have added this in our discussion section which now reads as follows:**

**“A potential approach to overcome these limitations could be to rely on information from causally derived targets beyond the causal evidence provided by earlier TMS studies (Cohen-Kadosh et al., 2008; Sack et al., 2009). For instance, studies have demonstrated that selecting targets from brain lesions or deep brain stimulation studies could potentially serve as effective TMS targets (see Siddiqi & Fox, 2023 for a review). While most causal targets have been derived from psychiatric symptoms such as depression or anxiety, examining behavioral deficits associated with focal brain injuries or deep brain stimulation can help selecting a TMS target for specific executive functions.” (page 31)**

(6) Could the authors please include a Methods figure detailing study procedures by visit?

**Response: A figure (Figure 1) has been added to the new version of the manuscript, including a consort diagram as well as the study procedure by visit.**

(7) I am curious about the authors’ speculations on their drift rate finding that TMS affected congruent versus incongruent trials. This seems worth briefly commenting on in the Discussion section. However, I leave this up to the authors if they feel the speculations are too speculative.

**Response: We agree with the reviewer that some speculation about the specificity of this change to the congruent trials is in order, although extensive discussion would probably not be appropriate, as the finding was unexpected and the study was not designed around testing it. The discussion now reads as follows:**

**“In the context of numerical Stroop task performance, rTMS to right IPL might be expected to increase the drift rate for both congruent and incongruent trials. This is based on the role of right parietal cortex in automatic spatial magnitude processing (in the case of the numerical Stroop task, a comparison of physical sizes) that Cohen-Kadosh et al. (2007) posited to explain their TMS findings. Right IPL rTMS should disrupt the automatic processing of number font size, leading to the subtracting out of its irrelevant contribution to the ongoing decision process. The decision process, having one less channel of useless information (i.e., noise) to integrate, is thus made more efficient, which should be reflected in an increased drift rate. In this** **interpretation for the cause of a change in drift rate with rTMS, the congruency condition should not matter. However, here only the congruent trials, but not the incongruent, showed this effect. A primary methodological difference between the Sack et al. and Cohen-Kadosh et al. studies and the present one is that subjects were relatively unpracticed on the task in the former but well-practiced in the latter, and the main difference in results was that an rTMS effect was seen directly in the RT data of the former studies but not the latter. In terms of the present drift rate finding, these differences suggest that within the decision process, at least in a well-practiced state, the mechanism underlying the integration of congruent properties differs and can be more easily distinguished from that underlying the integration of incongruent properties. With practice, the effect of right parietal rTMS on automatic processing seen in the earlier studies may change such that it only occurs in the congruent state, while the decision process in the incongruent condition may rely more heavily on other brain regions, such as anterior cingulate cortex. A further study comparing the effects of rTMS on subjects that are task-naïve vs. practiced would be needed to verify this. Moreover, the diffusion model results and speculations based on them result need to be taken cautiously as the analysis was done post hoc and the study was not designed optimally to evaluate the drift. For example, it only included 48 trials per condition, while it has been demonstrated that about hundred trials are needed to estimate this parameter robustly (Lerche et al., 2017). In addition, other models might be tested: for example, the drift diffusion model used here fits performance data to a single processing stage, while a dual-stage model, involving an early evidence accumulation phase followed by a later decision phase might exhibit a more robust fit to the data than a single-stage model (Hübner et al., 2010). Future studies might also want to conduct a drift modeling approach on single trial fMRI to delineate the neurocircuitry associated with practice and congruency effects.” (PAGE 22)**

# Reviewer #3:

This paper conducts a replication study of the facilitatory effects of rTMS on numerical Stroop task performance. They employed personalized targeting of the right intraparietal sulcus, as determined by peak activation. Although they were not able to replicate the reaction time results, they did find an increase in the drift rate in the congruent trials. The failure to replicate was attributed to various methodological differences, including task and stimulation administration. Overall, the paper is clearly and concisely written and the included figures illustrate the results in a simple and elegant way. I also applaud the authors for a replication attempt due to the profound lack of these studies. My main point of criticism is the employed statistical methods. The use of more robust statistical tools could give more confidence in the results by appropriately modeling the experimental structure. I am aware that this suggestion brings the scope of this study slightly past replication, and note that it already involves an innovative use of DDM. I believe the authors can enhance their already meaningful contribution to the literature.

**Response**: **We would like to thank the reviewer for sharing the concerns, and changed our statistical analyses from ANOVA to linear mixed model, more explanations are given in the response below.**

1) Introduction: #112: Please further motivate the decision to include the drift diffusion model (DDM) in the analyses. While I am aware that these models are highly influential and agree that they are interesting in this context, the theoretical reason to use it was not clear. I believe there are compelling reasons to do so, but they should be stated even if the authors did not have any clear or directional hypotheses.

**Response: We agree with the reviewer that the previous version of the manuscript did not provide enough theoretical reason to use the drift diffusion model that was chosen post-hoc. Therefore, we modified our introduction section with now reads as follows:**

**“Finally, we decided to perform a post-hoc behavioral modeling approach using a drift diffusion model to assess rTMS effects within a decision-making context. This model, by considering the distribution of reaction times rather than just central tendencies, offers more insight in decision making processes. By elucidating the dynamics of evidence accumulation over time, and by distinguishing between decision and non-decision processes, the model provides a more nuanced understanding of ongoing cognitive mechanisms. Drift diffusion models have demonstrated effectiveness in modeling performance data derived from simple tasks with short reaction times (< 1 s), as in the present case. In addition, they have been linked to neural behavior on a number of scales, from single cells to large populations (e.g., using EEG and fMRI), also highly relevant to the present study (Ratcliff, Smith, Brown, and McKoon, 2016). The drift diffusion models have also been successfully used to investigate the neuronal substrates for numerical representation using TMS (Cohen-Kadosh et al., 2010). Therefore, it was expected that this modeling approach would reveal more subtle rTMS effects, as recommended by Hartwigsen et al. (2015).” (PAGE 6)**

2) I also think that while the Stroop task is quite old and famous, the deck is stacked against some studies due to the relatively small and variable behavioral effect. The authors do discuss these issues, but I do think a bit more could be stated about using traditional TMS sequences to find small and noisy effects in general. For instance, is there a confidence interval in which we would expect the current and previous study’s results to fall?

**Response: While we acknowledge the Reviewer’s comments about the limitations of the numerical Stroop task, we set out to replicate findings using this task specifically, which had been significantly affected by rTMS manipulations in two previous studies. This gave us some confidence in finding our own effect, especially with the addition of extra behavioral practice on the task, which may have served to reduce the variability in the behavioral effect. In general, we agree with the Reviewer that care must be taken in task choice for TMS experiments, as most cognitive tasks exhibit a good degree of variability. The key in the choice is reliability. The congruency effect in the reaction time in the numerical Stroop task is quite robust both at the group level and at the individual level, as in the present study where all participants were slowed in the incongruent condition. The robustness of the congruency effect allows it to be used in behavioral experiments on decision making, manipulating variables such as the proportion of congruent to incongruent trials or the numerical distance between the two stimuli in individual trials (e.g., Shichel and Goldfarb, 2022). This sort of reliability in the congruency effect across conditions was found in the present study, where all subjects continued to demonstrate RT slowing in both active and sham rTMS conditions. We didn’t see a TMS effect on RT, but by incorporating the variability inherent in the task (by using a drift diffusion model which utilized the RT distribution), we were able to find a very strong TMS effect in which all participants but one showed an increase in drift rate in the congruent condition. This provided evidence that the lack of effect in the RT data may have been due to our addition of practice rather than to the inherent variability found in the task.**

3) Methods: Was motion confound regression applied to the BOLD time series? If so, please indicate which motion parameters were used.

**Response**: **Motion reduction (AKA correction) was included in the processing as follows for each dataset. First, the EPI volume with fewest outliers (the MIN_OUTLIER) was identified, which should be a stable way to select a reference volume for alignment. AFNI's 3dvolreg was then used to estimate rigid body motion across the EPI acquisition, and these six motion profiles (3 rotation and 3 translation) were included in the "regress" block GLM as regressors of no interest. Additionally, the Enorm (Euclidean norm) of the first differences of the motion profiles was calculated, providing a scalar measure of the amount of subject motion per TR. This was thresholded at 0.3 mm, to create remove time points of high motion from the GLM. Additionally, the number of outliers within the brain mask at each TR was calculated, and this "outlier fraction" was thresholded at 5%, creating another censoring measure to apply to the time series.**

4) Did the authors consider that the observations are not independent, as many samples are derived from the same participant? The use of a linear model, such as an ANOVA may not be appropriate in this case and may warrant the use of more advanced statistical methods such as a linear mixed effects model.

**Response: We did not consider that observations could not be independent, but in case it is, we decided to replace our ANOVA by a linear mixed model for the analysis of the rTMS effect. We also performed a linear regression analysis for the effect of covariates on rTMS effects.**

**The results section now reads as follows:**

**“A linear mixed model was performed with participants as a random effect, and Stimulation, Congruency and Order as fixed effects. The model revealed a main effect of Congruency (F(2, 25.30) = 47.56, p < 0.001), with incongruent trials slowing down participants (492 ± 15 ms) compared to congruent (439 ± 12 ms) and neutral trials (457 ms ± 12 ms) (p < 0.01 for each pair-wise comparison). The model also revealed a significant interaction with Stimulation, Congruency and Order (F(2, 44.43) = 3.68; p = 0.03). However, decomposition of this interaction with post-hoc Bonferroni-corrected contrasts did not reveal any significant difference between active and sham rTMS.” (PAGE 15)**

**And:**

**“In the first analysis, we performed a linear regression analysis with a backward selection method to tested the impact of several covariates that could impact rTMS effect (participant’s age, functional activations at the stimulated target, scalp-to-cortex distance, distance to Sack’s targets, and stimulation intensity, expressed as a percentage of the resting motor threshold, and number of days between MRI acquisition and TMS session) on rTMS effect [..].This method allowed to iteratively remove non-significant variables from the model, and thus to identify the most relevant predictors of rTMS effect while controlling for potential confounding variables. The final model showed that only stimulation intensity predicted the rTMS effect (F(1,13) = 13.09, p = 0.004, adjusted R2 = 48.2%). (PAGE 17-18)**

5) For the DDM, although 5% of the trials were modelled with a different distribution, were outlier reaction times (< 200ms) removed from the analyses? It is likely that these trials reflect a different underlying cognitive process than what is trying to be measured.

**Response: We agree with the reviewer that this could reflect a different mechanism, however in this sample the fastest of the observed reaction times in was 230ms so we did not have to remove them from the analysis.**

6) There are concerns that the number of trials used for each of the DDMs may have been too low, which may have affected the precision of parameter estimations. Please refer to Lerche et al. 2017 and comment with regards to the current study.

**Response: We agree with the reviewer: our experiment included 24 trials of each Congruency per block, and only 2 blocks per stimulation condition leading to only 48 trials for each condition of interest, which is way below the recommended number. This has been added into the discussion section which now reads as follows:**

**“Moreover, the diffusion model results and speculations based on them result need to be taken cautiously as the analysis was done post hoc and the study was not designed optimally to evaluate the drift. For example, it only included 48 trials per condition, while it has been demonstrated that about hundred trials are needed to estimate this parameter robustly (Lerche et al., 2017).(PAGE 23)**

7) Results: As the lack of a size congruency effect was one of the main negative findings in the replication, please include a figure for the demonstrating the lack of a size congruency effect.

**Response**: **We agree with the reviewer and added the SCE figure to the results (Figure 3).**

8) Discussion: There have been multiple studies involving DDM in the context of attentional and cognitive control that have required modifications to the DDM to account for these types of data (Hübner et al., 2010). Please refer to these works in the limitations section.

**Response**: **We added this potential dual stage two phase model in the discussion section of our manuscript which now reads as follows: “In addition, other models might be tested: for example, the drift diffusion model used here fits performance data to a single processing stage, while a dual-stage model, involving an early evidence accumulation phase followed by a later decision phase might exhibit a more robust fit to the data than a single-stage model (Hübner et al., 2010).” (PAGE 23)**

9) Although the authors use the DDM results to discount the role of the right IPL in automatic spatial magnitude processing, do they have any cognitive or neurobiological explanation for why the drift rate may have increased in only the congruent condition?

**Response: We have expanded our previous discussion speculating on the specificity of this effect to the Congruent condition, as well as suggestions for future research to keep exploring the neurocircuitry underlying this result: “In the context of numerical Stroop task performance, rTMS to right IPL might be expected to increase the drift rate for both congruent and incongruent trials. This is based on the role of right parietal cortex in automatic spatial magnitude processing (in the case of the numerical Stroop task, a comparison of physical sizes) that Cohen-Kadosh et al. (2007) posited to explain their TMS findings. Right IPL rTMS should disrupt the automatic processing of number font size, leading to the subtracting out of its irrelevant contribution to the ongoing decision process. The decision process, having one less channel of useless information (i.e., noise) to integrate, is thus made more efficient, which should be reflected in an increased drift rate. In this interpretation for the cause of a change in drift rate with rTMS, the congruency condition should not matter. However, here only the congruent trials, but not the incongruent, showed this effect. A primary methodological difference between the Sack et al. and Cohen-Kadosh et al. studies and the present one is that subjects were relatively unpracticed on the task in the former but well-practiced in the latter, and the main difference in results was that an rTMS effect was seen directly in the RT data of the former studies but not the latter. In terms of the present drift rate finding, these differences suggest that within the decision process, at least in a well-practiced state, the mechanism underlying the integration of congruent properties differs and can be more easily distinguished from that underlying the integration of incongruent properties. With practice, the effect of right parietal rTMS on automatic processing seen in the earlier studies may change such that it only occurs in the congruent state, while the decision process in the incongruent condition may rely more heavily on other brain regions, such as anterior cingulate cortex. A further study comparing the effects of rTMS on subjects that are task-naïve vs. practiced would be needed to verify this.”** (**PAGE 22-23)**

10) The choice of fMRI preprocessing parameters can change the findings of otherwise identical studies. Please comment on the differences in preprocessing schemes and how they may have affected your results.

**Response**: **Indeed, the choice of processing will affect results to some extent. This was observed in the "31 variants" study by Pauli et al. (2016) and commented on in the NARPS results by Botvinik-Nezer (2020). Note that one of the most important things done here to mitigate differences that might arise due to processing was to use more complete result reporting, and to reduce dichotomization that makes results unstable and more sensitive to processing choices. In particular, we used transparent thresholding in order to highlight results, as opposed to "hiding" many results away; this was first suggested by Allen et al. (2012) and was more recently shown to lead to much more informative comparisons of results across studies (Taylor et al. 2023)---in particular, showing how the results across various teams in the NARPS study are actually quite similar and in strong agreement of results, simply with varying degree of strength.**

**In addition, here we have used a well-tested pipeline tool to build the processing which uses many tools that have been validated and tested over the years (afni_proc.py has existed and been widely used since 2006). For example, the pipeline uses AFNI's 3dREMLfit, which was found to be the best available software tool for temporal autocorrelation modeling (Olszowy et al. 2019); AFNI's 3dvolreg, which was found to provide the most accurate and least blurring motion correction (Oakes et al., 2005); EPI-anatomical alignment with AFNI's 3dAllineate and lpc+ZZ cost function, which has been shown to improve alignment across datasets with differing contrasts (Saad et al., 2009); 3dQwarp for nonlinear registration, which has performed well in comparison with other nonlinear alignment tools (Cox and Glen, 2013); and in general AFNI's resampling has been shown to be stable (Cox and Taylor, 2017), in comparison to other software (see Mueller et al., 2017). The processing parameters chosen were fairly standard (see Taylor et al., 2018, for more description of basic afni_proc.py options).**

12) Minor: #213: Was the intention “highest tolerable intensity”?

**Response: No, the intention was for the participant to feel the sensation but not for it to be painful.**

References:
Lerche, V., Voss, A., & Nagler, M. (2017). How many trials are required for parameter estimation in diffusion modeling? A comparison of different optimization criteria. Behavior Research Methods, 49(2), 513–537. <https://doi.org/10.3758/s13428-016-0740-2>
Hübner, R., Steinhauser, M., & Lehle, C. (2010). A dual-stage two-phase model of selective attention. Psychological Review, 117(3), 759–784. <https://doi.org/10.1037/a0019471>

# Reviewer #4:

General comments: Overall this is a valuable study for the cognitive TMS field. It highlights the many factors that influence whether a TMS effect on behaviour will be observed. The discussion is particularly strong in this regard.

**Response: Thank you for the useful comments. We provide below a point by point response to your concerns.**

1) The study is primarily posed, however, as a replication of Sack et al. (2008), looking at the efficacy of individualised fMRI targeting to modulate numerical Stroop task performance. The aim of the Sack study was to compare different targeting approaches, whereas here the aim was to replicate one of the results from Sack et al. My first suggestion is therefore to change the title of the paper, as the current study is not addressing the targeting issue directly. (If replication of the targeting approach was the main aim of the present study, then this would have been better achieved by comparing against other targeting approaches, as per the original study.)

**Response: We agree with the reviewer, and as this has been pointed out by most of the reviewers, we decided to rebrand the manuscript so that it does not read as a replication of Sack’s study but instead as a tentative of testing whether the same effect could be found with the new techniques that have been developed in the literature since then, while focusing only on the optimal targeting approach, individualized fMRI targeting.**

**The new title is: “Lessons learned from an fMRI-guided rTMS study on performance in a numerical Stroop task.”**

**The abstract now reads as:** “**The Stroop task is a well-established tool to investigate the influence of competing visual categories on decision making. Neuroimaging as well as rTMS studies have demonstrated the involvement of parietal structures, particularly the intraparietal sulcus (IPS), in this task. Given its reliability, the numerical Stroop task was used to compare the effects of different TMS targeting approaches (Sack et al., 2009), and elegantly demonstrated the superiority of individualized fMRI targeting. We performed the present study to test whether fMRI-guided rTMS effects on numerical Stroop task performance could still be observed while using more advanced techniques that have emerged in the last decade (e.g., electrical sham, robotic coil holder system, etc). To do so we used a traditional reaction time analysis and we performed, post-hoc, a more advanced comprehensive drift diffusion modeling approach. Fifteen participants performed the numerical Stroop task while active or sham 10 Hz rTMS was applied over the region of the right intraparietal sulcus (IPS) showing the strongest functional activation in the Incongruent > Congruent contrast. This target was determined based on individualized fMRI data collected during a separate session. Contrary to our assumption, the classical reaction time analysis did not show any superiority of active rTMS over sham, probably due to potential confounds such as potential cumulative rTMS effects, and the effect of practice. However, the modeling approach revealed a robust effect of rTMS on the drift rate variable, suggesting differential processing of congruent and incongruent properties in perceptual decision-making, and, more generally, illustrating that more advanced computational analysis of performance data can elucidate the effects of rTMS on the brain where simpler methods may not.” (PAGE 2)**

**We modified the introduction as follow: “Sack et al. (2009) concluded that the most effective approach was individualized targeting based on the task-related fMRI. This was an important result for the brain stimulation field in general, as one of the main moderators of rTMS efficacy is the targeting approach (Beynel et al., 2019). More than a decade after this elegant demonstration of the importance of individualized fMRI targeting, experimental TMS methodologies have advanced, with for example the development of robotic coil holders that allow for more precise and stable positioning of the coil relative to the stimulation target, and an electrical sham technique that mimics the TMS-induced sensations, and thus potentially better blinds subjects to experimental conditions. In the present study, we tested whether the addition of these techniques combined with an individualized fMRI-based targeting approach endorsed by Sack and colleagues could improve the rTMS effect on numerical Stroop performance, with the expectation based on Cohen Kadosh et al. (2007) and Sack et al. (2009) that rTMS applied over the IPS would decrease the interference of incongruent stimuli and thus lessen of the reaction time slowing in that condition. Additionally, contrary to the prior study from Sack et al., participants completed the task multiple times preceding rTMS application (one block of trials before the MRI session, 4 blocks during MRI, and one block in the TMS session prior to TMS), to stabilize performance and minimize variability due to practice effects. This approach enabled us to probe the influence of task practice on behavioral performance and fMRI activations. (PAGE 5)**

2) While the finding of targeting approach (individual v group fMRI clusters for example) might not have been directly replicated, this issue has come up in other publication (e.g., Feredoes et al., 2007, J Neurosci), and many have used an individualised fMRI approach and shown individual variability around a specific target. Alongside findings from e-field modelling, it’s not clear this is such an issue these days.

**Response:** **We agree that using individualized fMRI for TMS targeting has been used successfully for a few decades now, as has using group mean fMRI; and that a direct comparison has been performed in Feredoes et al. (2007). However, to our knowledge, determining the optimal number of trials/blocks in the fMRI design to attain sufficient statistical power while balancing the effects of practice, learning, and fatigue remains a challenge.** **In the revised version of the manuscript, we added a new analysis in the results that shows how brain activations changes over the four blocks of fMRI activation, which may explain the inter-subject topographical variability. The results section now reads as follows: “To evaluate whether the lack of an rTMS effect may have been due to targeting issues resulting from a change in fMRI activation across blocks, which may have resulted from practice, learning, and/or fatigue, we investigated how activations differed between the first and last pairs of blocks. Our results showed that while activation in the bilateral IPS was strong during the first two blocks, IPS activity vanished during the last two blocks (Figure 6). Given that these changes were observed while accuracy remained quite high and constant across all blocks, and while reaction time continued to improve (Figure 2), we suspect this change is due to a learning effect and hypothesize two potential mechanisms: 1) the IPS was highly involved in the earlier learning stages of the task and became less necessary with practice, with possible activity shifts to other regions; 2) the number of IPS neurons necessary for task performance decreased with the optimization caused by practice, with subsequent diminishment of signal. If correct, these hypotheses indicate the need for a better understanding of the local and network changes associated with task practice and learning effects to estimate the best targeting parameters. In the present case we potentially would have seen stronger rTMS effect if we only acquired two blocks of fMRI, and this may have served as a better target for rTMS if the latter of the two reasons stated above for the activation decrease was true.” (PAGE 19).**

3) While replications, especially in the cognitive TMS field, are always extremely valuable, the ‘message’ of this study is more related to the number of considerations that a researcher must face when designing and conducting study on behaviour. I would therefore suggest re-framing the paper along these lines, through an attempt to replicate what could be regarded as a robust finding, given the reliability of the Stroop effect and individualised targeting. I feel that this would be of interest to the field, given that the targeting of cognitive processes with TMS, while conceptually simple, remains challenging, and the targeting approach is only one consideration when it comes to maximising TMS efficacy.

**Response: We agree with the reviewer, and as mentioned in our response to your first comments we decided to reframe the manuscript so that it does not read as a replication approach.**

4) Additional comments: How were the contrast maps thresholded? The t-values of the stimulated targets in Table 1 seem on the low side? Could some more detail be provided on how the IPS target was selected? The methods states that the ‘statistical peak’ within the right IPS was chosen (line 191), but what if there was more than one peak? Was it the cluster with the highest t-value?

**Response: For the individualized targeting approach, the t-stat for each of the participants was loaded on the neuronavigation system, along with the IPS mask converted into native space. The threshold was first set at 1.96 (to only display significant activation in the Incongruent > Congruent contrast p = 0.05). If strong activations were found, threshold was increased to define the peak. However, if no significant activation was found threshold was decreased until activation was detected within the mask. For this reason, the t-value was low in some participants. The highest t-value was always selected if there was more than one peak.**

5) Were there any further anatomical constraints beyond being within the Neurosynth mask? From Figure 4 it does look like the individual targets are anatomically variable, which is not an issue by itself, but some of the targets look like they are in the precuneus, and several others are in the inferior parietal cortex.

**Response: No, unfortunately we only used the Neurosynth mask, and, as we agree with the reviewer that not using more anatomical constraints represents a limitation, we added that to our revised manuscript: “To select the TMS target, we used the IPS mask from the Neurosynth website (https://neurosynth.org/), within which we estimated the location the peak activation for each participant. However, the size of this mask was quite large and the choice of target withing this mask suffers from a large variability across subjects in the location of the target selected, probably due to the lack of additional anatomical constraints in our mask (Figure 2B).” (PAGE 30)**

6) Given the multiple parameters that comprise a TMS study, would it be worthwhile trying to combine as many of these as possible (since they were recorded) to see if they can predict an individual’s results? Currently the values from Table 1 were assessed as single correlations with behaviour. Could they instead be predictors in a regression model? (The distance to the Sack group coordinate might not be useful to include, however, as they did not demonstrate a significant effect of TMS when this was targeted.) Such an analysis would also fit with the aim of the paper, which is to highlight the need for more sophisticated analyses, but also it would fit with our understanding of individual differences in response to TMS.

**Response: Thank you for this comment. We agree, and added into our manuscript a regression analysis with a backward selection method to identify which of our covariates predicted the rTMS effect and replicated that only intensity did. This now reads as follows in the manuscript:**

**“In the first analysis, we performed a linear regression analysis with a backward selection method to test the impact of several covariates that could impact rTMS effect (participant’s age, functional activations at the stimulated target, scalp-to-cortex distance, distance to Sack’s targets, and stimulation intensity, expressed as a percentage of the resting motor threshold, and number of days between MRI acquisition and TMS session) on rTMS effect […] This method allowed to iteratively remove non-significant variables from the model, and thus to identify the most relevant predictors of rTMS effect while controlling for potential confounding variables. The final model showed that only stimulation intensity predicted the rTMS effect (F(1,13) = 13.09, p = 0.004, adjusted R2 = 48.2%). This suggests that participants who received the strongest stimulation intensity where the ones benefitting the most from rTMS (showing the fastest reaction times with active rTMS). We note that on average, in our sample, an intensity of 60% MSO led to a strong variability in stimulation intensity, which ranged from 94% to 188% rMT (mean = 128.9 ± 5.7% rMT, see Supp. Table 1 for individual participants data). None of the other variables: scalp-to-cortex distance, distance to Sack’s target, and number of days between MRI acquisition and TMS session, or fMRI activation at the stimulated target predicted the rTMS effect. (PAGE 17-18)**

7) For the group-level fMRI analysis, the lack of an effect in right IPS is not necessarily an issue, and would speak to the anatomical variability of this region. As mentioned above, this issue has been covered by others such as Feredoes et al. It may be worth highlighting this particular point in the discussion when mentioning the potential of left IPS as a better target.

**Response: We agree with the reviewer and added that in the discussion that now reads as follows: “Regarding the group fMRI results, contrary to our expectations, while we were able to find significantly activated voxels for most of our participants (64%), our results at the group level do not indicate any significant activations in the right IPS in the Incongruent>Congruent contrast. Instead, we found some small clusters in the left IPS, and some larger clusters in subcortical structures such as the corpus callosum, and the cingulate cortex, the latter regions involved in conflict monitoring (Botvinick et al., 2001). Besides, the lack of a group activation in the right IPS does not necessary imply that it was not a good target. Indeed, it has been demonstrated that fMRI group analysis might not always detect the brain regions that are necessary at the individual level, and instead consider the individual topographic variability as noise; and that applying rTMS on this subject-specific regions, even though variable, can lead to a significant rTMS effect while stimulating the group fMRI activation site does not (Feredoes et al., 2007). This has been added into the discussion section of our manuscript. (PAGE 31)**

# Reviewer #5:

I am reviewing “What is the best way to target TMS? Lessons learned from a replication attempt on fMRI-guided rTMS modulation of the numerical Stroop task.” by Beynel et al. In this study, the authors sought to replicate results from the influential Sack et al. 2009 paper, which showed the relative advantage and increase in statistical power obtained when using individualized vs group-MRI based (or scalp based) TMS targeting for improving performance in a numeric Stroop task. The current study largely failed to replicate Sack et al., and thoughtfully acknowledged a few reasons why that might be, while making recommendations for future studies. I appreciated the overall effort and study, and thought the manuscript was thoughtful and well written. However, some of the results, design and analysis choices, raised questions and a few concerns for me, which are delineated below:

**Response: We would like to thank the reviewer for their positive words on our manuscript and hope that our point-by-point response below will answer their concerns.**

Major
1. Most strikingly, from the way the intro was framed, I was under the impression that the authors were going to directly compared & contrast different TMS targeting strategies (e.g. anatomical vs. functional based) within subjects (in contrast with the between-subjects approach used by Sack et al.) However, the study design only included the individualized TMS targeting condition, as opposed to the critical comparison of e.g. MRI/group- based coordinates vs. individualized, functional-based coordinates. While this point might appear moot in the context of the current (null) results, I still think this point (i.e., the primary study aim, which is not exactly a replication of Sack et al., which would have required the aforementioned explicit targeting strategy comparison) should be recontextualized and clarified in the Intro (and title).

**Response: Thank you for raising that point, we agree with the reviewer and as this has been pointed out by all reviewers we rebranded our manuscript so that it does not read as a replication of Sack’s study but instead as a tentative of testing whether the same effect could be found with the new techniques that have been developed in the literature since then, while focusing only on the optimal targeting approach, individualized fMRI targeting.**

**The new title is: “Lessons learned from an fMRI-guided rTMS study on performance in a numerical Stroop task.”**

**The abstract now reads as:** **“The Stroop task is a well-established tool to investigate the influence of competing visual categories on decision making. Neuroimaging as well as rTMS studies have demonstrated the involvement of parietal structures, particularly the intraparietal sulcus (IPS), in this task. Given its reliability, the numerical Stroop task was used to compare the effects of different TMS targeting approaches (Sack et al., 2009), and elegantly demonstrated the superiority of individualized fMRI targeting. We performed the present study to test whether fMRI-guided rTMS effects on numerical Stroop task performance could still be observed while using more advanced techniques that have emerged in the last decade (e.g., electrical sham, robotic coil holder system, etc). To do so we used a traditional reaction time analysis and we performed, post-hoc, a more advanced comprehensive drift diffusion modeling approach. Fifteen participants performed the numerical Stroop task while active or sham 10 Hz rTMS was applied over the region of the right intraparietal sulcus (IPS) showing the strongest functional activation in the Incongruent > Congruent contrast. This target was determined based on individualized fMRI data collected during a separate session. Contrary to our assumption, the classical reaction time analysis did not show any superiority of active rTMS over sham, probably due to potential confounds such as potential cumulative rTMS effects, and the effect of practice. However, the modeling approach revealed a robust effect of rTMS on the drift rate variable, suggesting differential processing of congruent and incongruent properties in perceptual decision-making, and, more generally, illustrating that more advanced computational analysis of performance data can elucidate the effects of rTMS on the brain where simpler methods may not” (PAGE 2)**

**We modified the introduction as follow: “Sack et al. (2009) concluded that the most effective approach was individualized targeting based on the task-related fMRI. This was an important result for the brain stimulation field in general, as one of the main moderators of rTMS efficacy is the targeting approach (Beynel et al., 2019). More than a decade after this elegant demonstration of the importance of individualized fMRI targeting, experimental TMS methodologies have advanced, with for example the development of robotic coil holders that allow for more precise and stable positioning of the coil relative to the stimulation target, and an electrical sham technique that mimics the TMS-induced sensations, and thus potentially better blinds subjects to experimental conditions. In the present study, we tested whether the addition of these techniques combined with an individualized fMRI-based targeting approach endorsed by Sack and colleagues could improve the rTMS effect on numerical Stroop performance, with the expectation based on Cohen Kadosh et al. (2007) and Sack et al. (2009) that rTMS applied over the IPS would decrease the interference of incongruent stimuli and thus lessen of the reaction time slowing in that condition. Additionally, contrary to the prior study from Sack et al., participants completed the task multiple times preceding rTMS application (one block of trials before the MRI session, 4 blocks during MRI, and one block in the TMS session prior to TMS), to stabilize performance and minimize variability due to practice effects. This approach enabled us to probe the influence of task practice on behavioral performance and fMRI activations. (PAGE 5)**

2. A critical aspect of the results that should be taken into account explicitly across all analysis (and it currently isn’t) is the 3-way interaction with TMS order (sham first vs control first). If I followed the results correctly, the result obtained with the group who had sham first is precisely the predicted result. Given that TMS (sham vs. active) order clearly matters here, all subsequent analysis should include order in the model as either a covariate or perhaps more ideally as an interacting factor (including the SCE analysis!).

**Response: Thank you for raising that point, we added in the revised manuscript a new analysis that adds the Stimulation order in the SCE analysis. However, for the linear regression investigating potential covariates, and the rest of the exploratory analysis, we could not add it given the limited number of subjects in each group.**

**The methods now reads as follows: “The third analysis was conducted to replicate Sack et al.’s finding on the size congruency effect (SCE), the difference between reaction time for Incongruent and Congruent trials, to test whether active rTMS would decrease the SCE compared to sham stimulation. We also investigated whether rTMS impacted the facilitatory and inhibitory component of the SCE. For each of these analyses, we performed an ANOVA with Stimulation (active, sham) as a within-subject factor, and Order of stimulation (active first, or sham first) as a between-subject factor”. (PAGE 12)**

**The SCE results read as follows: “To replicate the findings of Sack et al. (2009), we calculated the SCE by measuring the reaction time (RT) difference between congruent and incongruent trials [SCE = RT (incongruent) - RT (congruent)]. We excluded data from an additional participant whose size congruency values exceeded two standard deviations above the group mean, analyses were therefore performed on 13 participants. Contrary to expectation, the repeated measures ANOVA did not reveal a main effect of Stimulation (F(1,11) < 1, p = 0.40: Active = 51 ± 16 ms; Sham = 46 ± 9 ms), Order (F(1,11) < 1, p = 0.58) or interaction between Order and Stimulation (F(1,11) < 1, p = 0.58), suggesting that contrary to Sack et al., rTMS in our study did not impact the size congruency effect (Figure 3). Following the methodology employed by Sack et al., we also calculated the facilitatory component as [RT (neutral) - RT (congruent)] and the interference component computed as [RT (incongruent) – RT (neutral)]. For the facilitatory component, neither the interaction between Stimulation and Order, nor the main effects reached statistical significance. However, for the interference component, a significant interaction between Stimulation and Order was observed (F(1, 11) = 9.39; p = 0.011, η^2^ = 0.21). However, post hoc analysis did not reveal any significant differences among pairwise comparisons (Active-first group: Active = 47 ± 26 ms, Sham = 21 ± 16 ms; Sham-first group: Active = 24 ± 17 ms, Sham = 36 ± 17 ms). The absence of broader significant findings may be attributable to an insufficient sample size, which could diminish the power of our analysis.” (PAGE 16)**

3. Relatedly, how does overall Stroop performance compare between Sack et al. the current study? I am finding that RT differences for Congruent vs. Incongruent were slightly larger in the Sack study (104ms, potentially providing a better range to modulate those effects) vs the current study (71ms?), but I am not finding the average accuracy data in the Sack study... That would be important to know and compare across the samples.

**Response: The reviewer is correct that the size congruency effect was larger in Sack’s study than in ours, and that it could allow for more changes with rTMS. Unfortunately, Sack does not report their accuracy data, so we cannot compare our accuracy to theirs - However, we expect that, such as for our data, the accuracy might have been high in his study as well. We initially thought that the difference could be explained by the greater amount of practice our participants had. However, even during the first block of practice, our SCE was lower than Sack with 62ms only. We instructed participants to respond as quickly and as accurately as possible, and there is a potential tradeoff between accuracy and RT that differs between the two studies. However, without knowing the average accuracy for Sack’s study, we cannot be sure.**

4. And how does the age of the participants compare? (I was struck by the relatively older age of the current sample, 39 y old). I am revisiting the Sack paper, and seeing that the mean age was 24 y old in that study… This point relates back to the performance point above, particularly if performance differs across the 2 samples… Does age correlate with Stroop performance and/or with the magnitude of TMS effects found here?

**Response: This is a good point, and the reviewer is right that our average age is greater than the average reported by Sack. Our volunteers ranged in age from 22 to 58 years old at the time of consent with an average age of 39 while the volunteers in Sack’s study were, on average, 24 years of age. All participants underwent brief neurological exams and were deemed cognitively healthy for the study, however past work using various Stroop tasks has identified age effects in task performance (e.g., Wolf et. al., 2014). However, most of these results identify performance deficits in individuals older than our volunteers (i.e., 60 + years of age). To test for a potential effect of age on our results, we first correlated age with performance during the practice blocks; and we also added age into the regression model testing potential predictors of rTMS effects. We found no significant effects of age in any of them, as described below and in the revised manuscript.**

**For the Stroop performance, the manuscript reads as follows: “In addition, as our group of participants was older (39 years old on average) than the participants in Sack’s study (24 years old on average), we investigated whether there was any correlation between age and reaction times in Congruent and Incongruent trials collapsed across the six practice blocks. Results did not reveal any significant correlations between them (Congruent trials: r = 0.26; p = 0.40; Incongruent trials: r = 0.45, p = 0.13), suggesting that age did not impact performance. (PAGE 14)**

**For the rTMS effect: “In the first analysis, we performed a linear regression analysis with a backward selection method to test the impact of several covariates that could impact rTMS effect (participant’s age, functional activations at the stimulated target, scalp-to-cortex distance, distance to Sack’s targets, and stimulation intensity, expressed as a percentage of the resting motor threshold, and number of days between MRI acquisition and TMS session) on rTMS effect […] This method allowed to iteratively remove non-significant variables from the model, and thus to identify the most relevant predictors of rTMS effect while controlling for potential confounding variables. The final model showed that only stimulation intensity predicted the rTMS effect (F(1,13) = 13.09, p = 0.004, adjusted R2 = 48.2%).” (PAGE 17-18).**

5. How far apart were the MRI and TMS sessions? Does the interval matter in terms of the magnitude of the effects?

**Response: MRI and TMS sessions were quite far apart in our sample and ranged from 8 to 174 days with an average of 89 days. We added this covariate in our new regression analysis and did not find any impact of this covariate (p = 0.89)**

6. Accuracy seemed quite a bit lower for the TMS day (88%) compared to the fMRI day (98%). Was that a significant difference between those days? If so, is it possible that TMS is impacting accuracy (and/or differing by TMS site)?

**Response**: **Accuracy was indeed lower on the TMS day than on the fMRI day; however, for several reasons performance on the different days is not directly comparable. In the TMS session a third condition (i.e., neutral) was introduced into the task, and the addition of a third stimulus condition can alter expectancies and set in ways that can alter performance. In addition, the lab environment was different between the MRI center and our TMS lab. Subjects changed position from lying down to a sitting posture. Unlike in the MRI session, rTMS was applied while subjects performed the task, which can be distracting. All of these elements can affect performance accuracy. Nevertheless, the expected pattern of responses remained constant between the two sessions, for example with subjects more accurate in the congruent condition than in incongruent. As we did not see a difference in accuracy between TMS conditions (i.e., active vs sham), and given that accuracy was lower during the task than during the practice block preceding TMS, it could suggest that the sensation and sound induced by stimulation, independently of whether it was real or placebo stimulation, could have acted as a distractor.**

7. I was wondering about whether there is a chance that the ‘sham’ condition in this study was less of a sham than what was expected…. which is a point the authors themselves raised in the Discussion. Is there a way to more directly get at this question, e.g., by comparing stroop performance (accuracy or RT) in the sham condition vs. the baseline fMRI day?

**Response: The reviewer is right that the sham condition might have been more active than expected. It is possible that the weak electrical current passed through the electrodes could have induced some neuronal changes that could have impacted performance. However, while it would be a good idea to compare the TMS sham condition to the MRI performance this is not possible with our current design. Indeed, the environment was highly different between those two sessions, which can impact performance; and more importantly the tasks differed significantly with the absence of neutral trials in the MRI session leading to a lack of task switching and inherently distinct performance characteristics in the two sessions, as reflected by the overall decrease in accuracy during the TMS visit.**

8. I was not entirely clear on the interpretation of Figure 3, particularly regarding the specificity to the Congruent condition. Can that interpretation be more clearly spelled out, both as a short summary in the Results section, as well as in the Discussion section?

**Response: We agree with the reviewer that we have not given enough interpretation on Figure 3. We therefore modify our results and discussion, and we hope that our changes made it easier to understand.**

**“The drift diffusion model revealed a significant interaction between Congruency and Stimulation (F(2,70)=10.9, p<.001, Fig 4). Post hoc comparisons revealed that while no difference were observed for incongruent (t(14)=0.90, p=.39) and neutral trials (t(14)=0.89, p=.39), the drift rate was significantly higher with active rTMS compared to sham stimulation for the congruent trials (t(14)=6.5, p < 0.001). While we would have expected rTMS to the right IPS to increase the drift rate for both congruent and incongruent trials, given its role in automatic spatial magnitude processing; the current finding suggests that within the decision process, at least in a well-practiced state, the mechanism underlying the integration of congruent properties differs and can be more easily distinguished from that underlying the integration of incongruent properties”. (PAGE 17)**

**And the discussion section now reads as follows: “However, here only the congruent trials, but not the incongruent, showed this effect. In the context of numerical Stroop task performance, rTMS to right IPL might be expected to increase the drift rate for both congruent and incongruent trials. This is based on the role of right parietal cortex in automatic spatial magnitude processing (in the case of the numerical Stroop task, a comparison of physical sizes) that Cohen-Kadosh et al. (2007) posited to explain their TMS findings. Right IPL rTMS should disrupt the automatic processing of number font size, leading to the subtracting out of its irrelevant contribution to the ongoing decision process. The decision process, having one less channel of useless information (i.e., noise) to integrate, is thus made more efficient, which should be reflected in an increased drift rate. In this interpretation for the cause of a change in drift rate with rTMS, the congruency condition should not matter. However, here only the congruent trials, but not the incongruent, showed this effect. A primary methodological difference between the Sack et al. and Cohen-Kadosh et al. studies and the present one is that subjects were relatively unpracticed on the task in the former but well-practiced in the latter, and the main difference in results was that an rTMS effect was seen directly in the RT data of the former studies but not the latter. In terms of the present drift rate finding, these differences suggest that within the decision process, at least in a well-practiced state, the mechanism underlying the integration of congruent properties differs and can be more easily distinguished from that underlying the integration of incongruent properties. With practice, the effect of right parietal rTMS on automatic processing seen in the earlier studies may change such that it only occurs in the congruent state, while the decision process in the incongruent condition may rely more heavily on other brain regions, such as anterior cingulate cortex. A further study comparing the effects of rTMS on subjects that are task-naïve vs. practiced would be needed to verify this” (PAGE 22-23).**

Minor
1. I found Figures 2 & 3 hard to parse as the Stroop effect is most easily visualized by comparing Congruent vs. Incongruent conditions directly, which are currently plotted far away from one another; wouldn’t plotting Congruent vs. Incongruent side-by-side, and Sham vs. TMS (etc) on the X axis, make those plots easier to read?

**Response: With our new linear mixed model to examine rTMS effect, while we found the interaction between Stimulation, Congruency and Order; the post-hoc analyses of the differences between active and sham rTMS were not significant. Therefore, we decided to remove figure 2 from our manuscript. Regarding figure 3 (which is now figure 4) we agreed with the reviewer that this is not the usual way to plot the congruency effect, however since our goal in the figure was to demonstrate the rTMS effect we decided to plot it this way.**

# References:

**+ Allen EA, Erhardt EB, Calhoun VD (2012). Data visualization in the neurosciences: overcoming the curse of dimensionality. Neuron 74(4):603-8.**

**+ Botvinik-Nezer R, et al. (2020). Variability in the analysis of a single neuroimaging dataset by many teams. Nature 582(7810):84-88.**

**+ Cox RW, Glen DR (2013). Nonlinear warping in AFNI. Presented at the 19th Annual Meeting of the Organization for Human Brain Mapping.**

**+ Cox RW, Taylor PA (2017). Stability of spatial smoothness and cluster-size threshold estimates in FMRI using AFNI. arXiv:1709.07471 [stat.AP]**

**+ Feredoes E, Tononi G, Postle BR. The neural bases of the short-term storage of verbal information are anatomically variable across individuals. Journal of Neuroscience. 27(41), 11003-11008.**

**+ Hübner, R., Steinhauser, M., & Lehle, C. (2010). A dual-stage two-phase model of selective attention. Psychological Review, 117(3), 759–784.**

**+Lerche, V., Voss, A., & Nagler, M. (2017). How many trials are required for parameter estimation in diffusion modeling? A comparison of different optimization criteria. Behavior Research Methods, 49(2), 513–537.**

**+ Mueller K, Lepsien J, Möller HE, Lohmann G (2017). Commentary: Cluster failure: Why fMRI inferences for spatial extent have inflated false-positive rates. Front Hum Neurosci 11:345.**

**+ Oakes TR, Johnstone T, Ores Walsh KS, Greischar LL, Alexander AL, Fox AS, Davidson RJ (2005). Comparison of fMRI motion correction software tools. Neuroimage. 28(3):529-543. doi:10.1016/j.neuroimage.2005.05.058**

**+ Olszowy W, Aston J, Rua C, Williams GB (2019). Accurate autocorrelation modeling substantially improves fMRI reliability. Nature Communications 10, 1220. doi.org/10.1038/s41467-019-09230-w**

**+ Pauli R, Bowring A, Reynolds R, Chen G, Nichols TE, Maumet C (2016). Exploring fMRI Results Space: 31 Variants of an fMRI Analysis in AFNI, FSL, and SPM. Front Neuroinform 10:24.**

**+ Saad ZS, Glen DR, Chen G, Beauchamp MS, Desai R, Cox RW (2009). A new method for improving functional-to-structural MRI alignment using local Pearson correlation. Neuroimage 44 839–848. doi: 10.1016/j.neuroimage.2008.09.037.**

**+ Shichel, I., & Goldfarb, L. (2022). The effect of proportion manipulation on the size-congruency and distance effects in the numerical Stroop task. Memory & Cognition, 50(7), 1578-1589.**

**+ Taylor PA, Chen G, Glen DR, Rajendra JK, Reynolds RC, Cox RW (2018). FMRI processing with AFNI: Some comments and corrections on ‘Exploring the Impact of Analysis Software on Task fMRI Results’. bioRxiv 308643; doi:10.1101/308643**

**+ Taylor PA, Reynolds RC, Calhoun V, Gonzalez-Castillo J, Handwerker DA, Bandettini PA, Mejia AF, Chen G (2023). Highlight Results, Don’t Hide Them: Enhance interpretation, reduce biases and improve reproducibility. Neuroimage 274:120138. doi: 10.1016/j.neuroimage.2023.120138**
